# Anatomical Variations of the Tibialis Anterior Tendon Insertion: An Updated and Comprehensive Review

**DOI:** 10.3390/jcm10163684

**Published:** 2021-08-19

**Authors:** Nicol Zielinska, Richard Shane Tubbs, Friedrich Paulsen, Bartłomiej Szewczyk, Michał Podgórski, Andrzej Borowski, Łukasz Olewnik

**Affiliations:** 1 Department of Anatomical Dissection and Donation, Medical University of Lodz, 90-419 Lodz, Poland; nicol.zielinska@stud.umed.lodz.pl (N.Z.); bartlomiej.szewczyk@umed.lodz.pl (B.S.); 2 Department of Neurosurgery, Tulane University School of Medicine, New Orleans, LA 70112, USA; shane.tubbs@icloud.com; 3 Department of Neurosurgery and Ochsner Neuroscience Institute, Ochsner Health System, New Orleans, LA 70112, USA; 4 Department of Anatomical Sciences, St. George’s University, 38-902, Grenada; 5 Institute of Functional and Clinical Anatomy, Friedrich Alexander University Erlangen-Nürnberg, 91054 Erlangen, Germany; friedrich.paulsen@fau.de; 6 Department of Topographic Anatomy and Operative Surgery, Sechenov University, 119991 Moscow, Russia; 7 Department of Diagnostic Imaging, Polish Mother’s Memorial Hospital—Research Institute, 90-419 Łódź, Poland; chilam@o2.pl; 8 Orthopaedics and Pediatric Orthopaedics Department, Medical University of Lodz, 90-419 Łódź, Poland; aborowski@xl.wp.pl

**Keywords:** tibialis anterior muscle, tendon rupture, new classification, diagnosis, fetuses, systematic review

## Abstract

The tibialis anterior muscle originates on the lateral condyle of the tibia, on the upper two-thirds of the lateral surface of this bone, on the anterior surface of the interosseous membrane and on the deep surface of the fascia cruris. The distal attachment is typically at the medial cuneiform and first metatarsal. However, the tibialis anterior tendon can vary morphologically in both adults and fetuses. Different authors have created new classification systems for it. The main aim of this review is to present condensed information about the tibialis anterior tendon based on the available literature. Another aim is to compare classification systems and the results of previous studies.

## 1. Introduction

The leg is divided into an anterior, a lateral and a posterior crural compartment [1]. The tibialis anterior muscle (TAM), also called tibialis anticus, belongs to the anterior compartment formed by the tibialis anterior, extensor hallucis longus, extensor digitorum longus and fibularis tertius [1]. It arises from the lateral condyle of the tibia and from the upper two-thirds of the lateral surface of the tibia. It also attaches to the anterior surface of the membrana interossea and to the deep surface of the fascia cruris. Its distal attachment (tendinous part) is on the medial cuneiform bone and the first metatarsal bone [1]. The tibialis anterior motor neurons are located in the ipsilateral L4 and L5 spinal ganglia [2]. The deep fibular (peroneal) nerve is responsible for motor innervation to the TAM [2]. The anterior tibial artery supplies blood to the muscle proximally, and the medial tarsal arteries (branches of the dorsalis pedis artery) supply the tibialis anterior tendon (TAT) distally [3,4].

The TAM plays an important role in dorsiflexion and inversion of the foot, which is important for maintaining balance [5] and for clearing and lowering the foot during the swing phase of gait [5,6,7]. The TAM also plays a key role in energy absorption during walking [6].

Tibialis anterior tendon rupture is a rare injury [7]. More injuries of this kind were observed when interest in physical activity by non-professional athletes grew [8,9,10]. Palpation examination is a basis for the diagnosis of a complete tendon rupture. However, further treatment requires confirmation by imaging. Palpation may be insufficient in cases of parietal tears, bursitis or tendinopathy. In these cases, diagnostic imaging, such as ultrasound (USG) with colour Doppler, computed tomography (CT) or magnetic resonance imaging (MRI), should be initiated [11,12].

The insertion of the TAT is morphologically variable, as confirmed by several studies [12,13,14,15,16]. Some authors have classified this muscle into distinct types, allowing various classification systems to be created [12,13,17]. The most common variant is the occurrence of one additional band. Sometimes two or more additional insertions were observed [1,2,9]. There are morphological variations not only among adults but also among fetuses [13].

The main aim of this review is to present condensed information on the TAT based on the available literature. Another aim is to compare the classification systems and the results of previous studies in both adults and fetuses. Information on the most common pathologies associated with TAT is also provided in this manuscript.

## 2. Tibialis Anterior Tendon among Adults

Anatomically, as mentioned above, the TAT inserts into the medial cuneiform bone and the first metatarsal bone [1]. However, subsequent studies have shown that this muscle can vary morphologically in both origin and insertion [12,13,14,15,16,17,18]. Although the variations associated with the proximal attachment are interesting, we want to focus on the distal attachment.

The TAT was first classified by Musiał [15] in 1963. He distinguished four types based on the number of prominent ligaments, placement of the insertion and differences in width. Type I, with a prevalence of 37.7%, had the tendon portion divided into two distal attachments of equal size, one at the medial cuneiform and the other at the base of the first metatarsal. Type II was also characterised by two ligaments, but they differed in width. The wider component was distal to the medial cuneiform bone and the narrower component was at the base of the first metatarsal. This was the most common Type, frequency 56.5%. Type III, with a frequency of 4.1%, had a tendinous component divided into a broad attachment (located at the medial cuneiform bone) and some thin fibres attached to the base of the first metatarsal. The courses of the two ligaments in type IV (1.7%) were similar to Type II, but the narrower portion in Type IV attached to the medial cuneiform bone and the broader portion attached to the base of the first metatarsal. Arthornhurasook and Gaew Im [16] in 1990 also studied anatomical variations of the TA tendon insertion. They distinguished three types of TAT. The courses of Type I (56.8%, the most common) and Type II (27.3%) were identical to the types extracted by Musiał [15] (Types I and II, respectively). During this research a new type of TAT was noticed (prevalence 15.9%), characterised by a single slip inserted on to the medial cuneiform only [16]. The next study of morphological variants of the TA tendon was conducted by Brenner [17]. Five types of TAT were distinguished. Three of them (Type I, 27.6%; Type II, 45.5%; Type III, 25.6%) were consistent with the first three types described by Musiał [15], and the next type (1.3%) was identical with the new type described by Atrhornhurasook and Gaew Im [16]. Brenner also identified a type (1.9%), represented by one single band, the distal attachment of which was located on the first metatarsal. Another scientist—Willeggar et al. [14]—published results based on the classification system by Musiał [15]. Although the courses of Type I (48.8%), Type II (48.8%), and Type IV (2.4%) (Type III was absent in this study) were identical to these described by Musiał [15]. Willeggar divided Type I into two subtypes because the morphological appearance of the TAT footprint in this type was heterogeneous. Subtype Ia (7.3%) was characterised by a wide insertion on to the medial cuneiform and first metatarsal, and subtype Ib (41.5%) was characterised by a narrow insertion on to the same places.

The most recent classification, created by Olewnik et al. [12], somewhat resembled a combination of the classifications by Musiał [15] and Arthornhurasook and Gaew Im [16]. Anatomical dissection allowed five types to be distinguished, four of them consistent with the four types distinguished by Musiał [15]. The fifth type was characterised by a single band inserted on to the medial cuneiform bone, identical to the new type described by Arthornhurasook and Gaew Im. [16]. The important thing is that in the classification by Olewnik et al. [12], Type III was represented by a wider component that inserted onto the first metatarsal and a narrower component that inserted onto the medial cuneiform, classified as Type IV in previous studies by Musiał [15], Brenner [17] and Willeggar et al. [14]. Similarly, Type IV distinguished by Olewnik et al. [12] was Type III in the Musiał [15] and Willegram et al. [14] classification systems. The next difference is that in the Olewnik et al. study [12], this type was represented by three tendons (one inserted on to the medial cuneiform bone and two on to the first metatarsal); in the Musiał study [15] it was characterised by a tendinous part divided into one wide insertion (on the medial cuneiform) and some thin fibers attached to the base of the first metatarsal. In the study by Willeggram et al. [14], none of the specimens had this type. To make things easier, we will only use the Olewnik et al. classification system [12] in the remainder of this article.

Interestingly, Olewnik et al. [12] divided their research into two parts: an anatomical part, which included the anatomical preparation of the TAT in cadavers, and a sonographic part, which included the USG examination in human subjects. This study is therefore the first systematic classification of the TAT based on an anatomical study but partially confirmed by sonography. The anatomic study was performed on one hundred lower limbs (50 paired, 62 male, 38 female) fixed in 10% formalin solution before examination. The mean age “at death” of the cadavers was 63.8 years. The first stage of dissection was removing the skin and superficial fascia to the crural fascia of the leg. Next, the skin and subcutaneous tissue were removed. Then muscular structures were cleaned and morphometric measurements were checked by an electronic digital caliper. Three morphometric parameters differed significantly between types of the TAT: TAT first band thickness at the point of insertion (mean value ranged from 2.12 mm to 3.24 mm, depending on the type), TAT first band width at the point of insertion (mean value ranged from 5.10 mm to 10.65 mm, depending on type), and distance to the origin of the first band (mean value ranged from the 129.94 mm to 159.79 mm, depending on type). In the sonographic study, 50 volunteers (23 women, 27 men) took part. Their mean age was 39 years (25–55). The patients were placed in a supine position with flexed hip and knee joints, and both feet were examined. The morphometric measurements included cross-section of all tendons of the TAM at their proximal attachment. The placement of the insertion was also assessed, and the results were surprising. Type IV, represented by a tendon branch attached to the medial cuneiform (one ligament) and to the first metatarsal (two ligaments—at the base and at the shaft/distal part), which was identified in the anatomical preparation, was not found in the ultrasound examination of the subjects. One possible reason is that the thin third ligament is only visible at very high ultrasound resolutions. In addition, the sonographic examination was able to distinguish a new type represented by two equal-sized ligaments, both attaching to the medial cuneiform bone. This is the first description of such a type of TAT in the literature. It occurred in 12% of the subjects examined.

**Figure 1:**
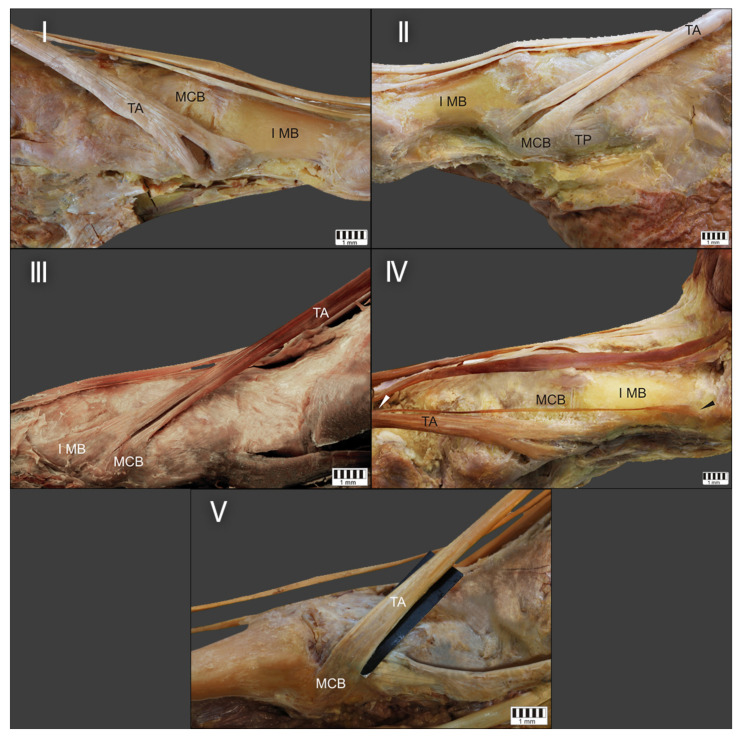
Tibialis anterior tendon. Classification by Olewnik et al. TA tibialis anterior, MCB medial cuneiform bone, I MB first metatarsal bone. (Type I—left foot; Type II, III, IV, V—right foot). Type I—the tendon splits into two equal-size bands that insert to the medial cuneiform bone and base of the first metatarsal. Type II—the tendon splits into two bands that insert to the medial cuneiform bone (larger component) and the base of the first metatarsal (smaller component). Type III—the tendon splits into two bands that insert to the medial cuneiform bone (smaller component) and base of the first metatarsal (larger component). Type IV—the tendon trifurcates, inserting to the medial cuneiform bone (one band) and the first metatarsal (two bands, to the base and the shaft /distal part). Type V—a single band inserts to the medial cuneiform bone.

**Figure 2:**
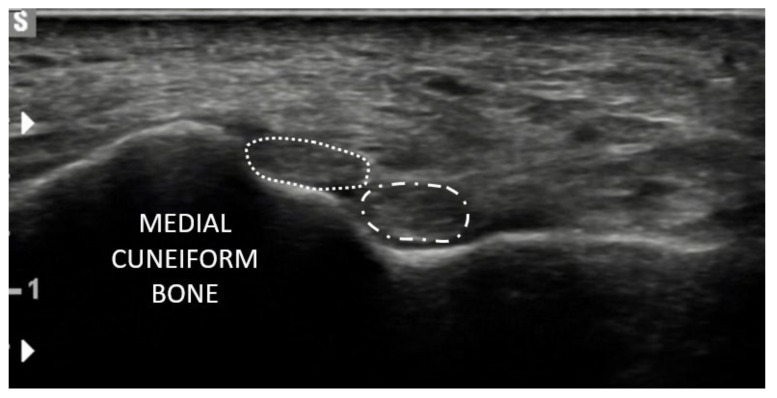
US examination. Type VI, consisted of two equal-sized ligaments, both attached to the medial cuneiform bone.

Summing up the most recent classification by Olewnik et al. [12] (which we will refer to in the next section of this overview), we can divide the morphological variations into six types. Type I is characterised by two insertions of equal size located on the medial cuneiform and the first metatarsal. Type II is represented by two bands, the wider one inserted on the medial cuneiform and the narrower one on the base of the first metatarsal; this was the most common type in the subjects in the sonographic study. Type III was characterised by the tendinous portion splitting into two ligaments, one inserted at the medial cuneiform (smaller portion) and the other at the base of the first metatarsal (larger portion). As mentioned above, type IV was only observed in the anatomical part of this study; no sonographically examined specimen showed this type. It was characterised by three tendons, one of which attached distally to the medial cuneiform bone, the other two to the base and shaft of the first metatarsal. Type V was characterised by a single ligament attached to the medial cuneiform bone, and this was the most common type among the dissected cadavers. The presence of Type VI was only detected on ultrasound examination; it consisted of two equal-sized ligaments, both attached to the medial cuneiform bone. All anatomical types identified by Olewnik et al. [12] are shown in Figure 1, and the USG type (VI) in Figure 2.

## 3. Tibialis Anterior Tendon among Fetuses

**Figure 3:**
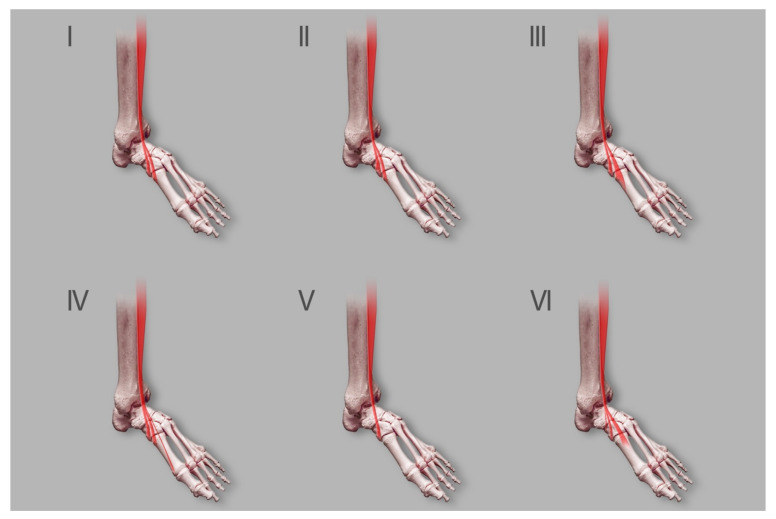
Classification by Karauda et al. [13]. Type I—the tendon splits into two equal-size bands that insert onto the medial cuneiform bone and base of the first metatarsal 1. Type II—the tendon splits into two bands that insert onto the medial cuneiform bone (larger component) and the base of the first metatarsal (smaller component). Type III—the tendon splits into two bands that insert onto the medial cuneiform bone (smaller component) and the base of the first metatarsal (larger component). Type IV—the tendon trifurcates, inserting to the medial cuneiform bone (one band) and the first metatarsal (two bands—to the base and the shaft /distal part). Type V—a single band inserts onto the medial cuneiform bone. Type VI—the tendon splits into three bands, onto the medial cuneiform bone (the smallest component) and base of the first metatarsal (the middle and larger components).

The insertion of the TAT was also researched among fetuses. Karauda et al. [13] created the first classification of this muscle among human fetuses (Figure 3). Another goal of the present study was to identify differences between this group and adults. Moreover, Karauda et al. [13] focused on determining whether some fetuses have additional TAM bands.

The authors examined one hundred lower limbs (fifty spontaneously aborted human fetuses aged 18–38 weeks of gestation). The results showed that TAT was present in all samples (48 lower limbs from 24 female fetuses and 52 from 26 male fetuses). Statistical analysis revealed no significant differences between the sexes in age, craniosacral length or total lower limb lengthThe next stage of this study was to create the first classification system of the TAT among fetuses based on morphological variations of its insertion. Karauda et al. [14] relied on the abovementioned classification system by Olewnik et al. [8]. The classification system for fetuses was based only on the anatomical part of the study of adults (characterised by five types) [8]. A new type of TAT was identified in fetuses; it has been called Type VI. Importantly, it is not the same as Type VI (two equal-sized ligaments attaching to the medial cuneiform bone) identified in the sonographic part of the study by Olewnik et al. [12]. In the fetuses, Type VI was characterised by the tendinous part of the TAM being divided into three distinct ligaments—Table 1. The distal attachment of the smallest component was at the medial cuneiform bone. The middle and larger components attached to the base of the first metatarsal. This type was found in four lower limbs (two right, two left). It was the basis for the extension of the previous classification. Although the fourth type (three distal attachments, one to the medial cuneiform, two to the first metatarsal (base and shaft)) was not found in this group, it was not removed from the classification. One possible reason for its absence could be that the group of fetuses was too small; Type IV could possibly be found in a larger population of fetuses. However, considering that the characteristic feature of Type IV is its three distal appendages, it is possible that the thinnest tendon has not yet developed in fetuses. Other morphological variations of the TAT in fetuses were similar to those in human adults. For example, Type I was characterised by a tendon component divided into two ligaments attached to the medial cuneiform bone and the base of the first metatarsal; and type II had the larger component attached to the medial cuneiform bone and the smaller component attached to the base of the first metatarsal. A similar morphological variation is also associated with Type III, which is characterised by a tendon component divided into two distal attachments located at the medial cuneiform bone (smaller component) and at the base of the metatarsal bone (larger component). The final example of a close resemblance between fetuses and adults is Type V, which had a single ligament attached to the medial cuneiform bone.

## 4. Descriptive Comparison between the Results of Fetal Anatomical Dissection, Adult Anatomical Dissection and Adult Sonographic Examination

Table 2 shows a descriptive comparison between the results of the fetal anatomical dissection, adult anatomical dissection and adult sonographic examination. This table was created based on these three studies as they all have the same classification system.

The results of this comparison show that regardless of whether the anatomical study was done on fetuses or adults, the most common type was Type V, the second most common was Type I, the third was Type II and the fourth in frequency of occurrence was Type III. There are also some differences between the adult and fetal anatomical studies. The first is that Type IV is not present in fetuses but has a prevalence of 2% in adult cadavers. The next is the presence of Type VI in fetuses (the basis for updating the classification system) and its absence in adults. There are also similarities between the anatomical study in fetuses and the sonographic study in 50 adult subjects. The results of both studies did not include any cases of Type IV. Although there was a Type VI in both studies, this type differed in description. Type VI in the fetuses was characterised by the smallest component being inserted at the medial cuneiform bone and the medium and larger components at the base of the first metatarsal. Type VI in adults was characterised by two equal-sized ligaments inserted at the medial cuneiform bone.

## 5. Tibialis Anterior Tendon Rupture

The TAT may be related with some pathologies. One example is tibialis anterior tendon rupture (TATR), which can occur spontaneously [19,20,21,22,23] or following trauma [24,25]. The male population over 45 years of age is predisposed to a spontaneous type of tendon rupture [20,21]. It is more likely when a tendinopathy coexists [26]. We can also distinguish risk factors for spontaneous TATR, such as gout, systematic lupus erythematosus and rheumatoid arthritis, which are all members of the group of rheumatic diseases. Diabetes mellitus is another risk factor [19,26,27,28,29]. Interestingly, there are documented cases of spontaneous rupture connected with recent onset psoriasis, or corticosteroid injection around the tarso-talar joint [30,31]. Traumatic ruptures can be caused by lacerations or blunt trauma, typically due to forced plantar flexion of the foot and ankle, of which there are case reports in the literature. For example, Rimoldi et al. [32] described a patient suffering from acute rupture of the TAT. This patient complained about pain in the medial region of the midfoot and moved with a stepped gait, which is a form of gait abnormality represented by foot drop or ankle equinus due to loss of dorsiflexion [32]. Another case report by Jerome et al. [27] described a patient with TATR associated with gout. After repair by non-absorbable suture, the leg was immobilized in a plaster cast [27]. Another interesting case was described by Kashyap and Prince [30]. Their patient suffered from diabetes mellitus, and spontaneous TATR occurred. In such a comorbid disease, TATR can be misdiagnosed and mistaken for a peroneal nerve deficit, because its symptoms are foot drop and stepped gait too and a peroneal nerve deficit is usually correlated with the diabetes mellitus. The treatment instituted depended on sliding tendon lengthening of the proximal portion of the TAT.

As mentioned above, TATR is rare and is characterised by initial intense pain and weakness of the foot, associated with difficulty with full weight bearing of the affected limb. Walking improves within an hour of the injury, but there are still problems with dorsiflexion. We usually notice a sinking of the foot and hyperextension of the toes. This symptom results from an attempt to compensate for the weakened function of the TAM. A complete tendon rupture can only be diagnosed by palpation examination [7,12]. A characteristic gap along the route of the tendon is palpated. The proximal stub can also be palpated, but at the level of the ankle region. Patients often suffer from pain in the ankle region and describe a “slapping” of the foot during walking. Usually, the dorsum of the foot is swollen and bruised [19,20]. However, these symptoms usually only occur with fresh injuries. Sometimes patients report to the doctor only after a longer period of time, so that the symptoms may differ from those described above. In such cases, other pathologies, such as chronic anterior compartment syndrome of the tibia or paresis of the peroneal nerve, should be ruled out [33]. The likelihood of TATR is very high when there is a clinical triad of “pseudotumour” in the anteromedial aspect of the ankle, weakness of dorsiflexion of the foot and loss of tendon contour (tendon not palpable during resisted dorsiflexion) [21].

Although complete rupture can be diagnosed only by palpation, this diagnosis should be confirmed by imaging techniques to implement future treatment. The gold standard for confirming TATR is ultrasonography [12]. Determination by Olewnik et al. [12] of the morphological variants of TAT using ultrasound and comparison of the results with those of anatomical dissection made it possible to create a new classification that could be important for treatment and in surgical interventions. However, we believe that before using this technique for these two purposes, the classification of the type of TAT should be verified during imaging. We propose a new study similar to that of Olewnik et al. [12], also divided into two parts: anatomical and sonographic. However, the cohort group would consist only of cadavers. First, a lower leg of a cadaver would be examined by ultrasound and the type determined. Then the lower leg of the same cadaver would be dissected anatomically and the type determined according to the existing classification system. The classifications of type by sonographic and anatomical examination on the same individual cadaver would then be compared. The results would show whether ultrasound is useful in determining type and whether it can be used in treatment and surgical procedures to improve their effectiveness. If the type compatibility were close to 100%, this would be a perfect result with which to investigate a relationship between the types of TAT and the treatment of tendon ruptures.

## 6. Treatment of Tibialias Anterior Tendon Rupture

Treatment of TATR can be either conservative or surgical [12,34,35]. New studies have shown that surgical treatment is superior. Surgical operation leads to better functional outcomes and lower complication rates than conservative treatment, regardless of factors such as age, functional status of patients before surgery, timeliness of the operation (immediate or delayed) and comorbidities [34]. Surgery depends on reconstruction of the TAT, which includes tendon transfer, repair of all muscle, or allograft augmentation, allows the correct anatomical position of this muscle to be restored and leads to restoration of the natural level of functioning [12,14]. The main aim of reconstruction is to re-establish ankle dorsiflexion and inversion [36,37]. In some cases of TATR, an interpositional autograft is necessary. Typically, structures such as the plantaris tendon, extensor digitorum longus, extensor hallucis brevis and achilles tendon are used for this [29,38]. Sometimes an anterolateral thigh flap can also be used for soft tissue reconstruction [35]. The advantages of this structure, such as consistent and unchanged anatomy, long pedicle, localization (good distance from the ablative site) and allowing a two-team approach, enable it to be used for this purpose [39,40]. Another advantage is the possibility of creating multiple skin flaps as an effect of recruiting different types based on a single pedicle, thus reconstructing the composite defect. The anterolateral thigh flap can be accepted as the ideal free flap structure for lower limb reconstruction due to its maximum reconstructive capacity and minimal donor site morbidity [40]. Moreover, this musculocutaneous structure is associated with a shorter operative time [41]. During reconstruction in TATR cases, a suture anchor or a bio-tenodesis screw are usually used. Conservative treatment can be instituted for elderly patients with low functional demands or suffering from comorbidities that significantly contraindicate surgery [36]. However, if there is tendinopathy that increases the risk of spontaneous tendon rupture, rehabilitation is the method of first choice. This usually involves a combination of one or more methods of eccentric training, kinesiology taping, isometric and stretching exercises, manual therapy, electrotherapy or exercises associated with improving control of the lumbar-pelvic area [42,43].

Although these methods are commonly available, a good detailed anatomical knowledge of the course of the TAT and its distal insertion is necessary to achieve high treatment effectiveness. The rehabilitation method should be selected based on the insertion site and the course and force vector of this tendon. As mentioned above, the classification systems differ depending on the distal approach of the TAT [14,15,16,17], but they are inconsistent and are characterised by imprecision about the placement of the distal insertion. In our opinion, the first classification that could help in the treatment of TATR was made by Olewnik et al. [12] who compared the results between anatomical dissection and ultrasound examination. They suggested that different types of distal approaches are associated with different force distributions in the foot and ankle regions. They also noted that different types could be related to different courses of surgeries [12].

## 7. Conclusions

The tibialis anterior tendon is morphologically variable in both adults and fetuses. Several classification systems are available in the literature, but only one was based on both anatomical dissection and ultrasound examination. This system could be used, for example, in the management of TAT rupture.

## Figures and Tables

**Table 1:** This table compares the results of the mentioned studies in relation to the classification system created by Olewnik et al. [12].

Type	Olewnik et al., 2019 [12]	Olewnik et al. [11] Ultrasonography	Willegger et al. [14]	Brenner [17]	Arthornhurasook & Gaew Im [17]	Musiał [15]
Type I	34%	20%	7.3%	27.6%	56.5%	37.7%
Type II	24%	35%	48.8%	45.5%	27.3%	56.5%
Type III	11%	13%	2.4%	25.6%	1.7%	-
Type IV	2%	-	-	-	-	4.1% *
Type V	32%	20%	-	1.3%	15.9%	-
Type VI **	-	-	-	1.9%	-	-

* In Musiał’s classification [15], this type was described as a tendinous part divided into one wide insertion (located on the medial cuneiform) and some thin fibers attached to the base of the first metatarsal. ** this type was not included in the classification system by Olewnik et al. [11].

**Table 2:** Comparison of TAT prevalence between anatomical study of fetuses, anatomical study of adults and sonographic study of adults.

Type	Karauda et al. (2020) [13]	Olewnik et al. (2019) [12]	Olewnik et al. (2019) [11]
	(A.S. of Fetuses)	(A.S. of Adults)	(S.S. of Adults)
I	19%	31%	20%
II	12%	24%	35%
III	5%	11%	13%
IV	0%	2%	0%
V	60%	32%	20%
VI *	4%	0%	12%

* type VI distinguished by Karauda et al. [13] differs from type VI distinguished by Olewnik et al. [12] during the sonographic study. A.S. anatomical study, S.S. sonographic study.

## Data Availability

Please contact authors for data requests (Ł.O.; email: lukasz.olewnik@umed.lodz.pl).
